# Supplementary material for: Physical and psychological health in intern paramedics commencing shift work: Protocol for an exploratory longitudinal study
Source: PLoS One. 2022 Dec 1;17(12):e0273113. doi: 10.1371/journal.pone.0273113 (PMC9714933; doi:10.1371/journal.pone.0273113)
Supplement: S1 Appendix — (DOCX) [file pone.0273113.s001.docx]

**S1 Appendix. Microbiome Checklist**

*Please answer the following questions at the time of providing this sample:*

**Are you taking any medications (prescription or over the counter?):**

 Yes

 No

**If yes, can you write the names and doses?**

**How many cups of caffeinated drinks are you consuming per day on average? (coffee, tea, energy drinks, etc)**

________ per day

**How recently have you used antibiotics?**

Currently taking

within the last week

1-2 weeks ago (last fortnight)

2-4 weeks ago (last month)

1-3 months ago

>3 months ago

**How recently have you consumed >4 standard drinks in one day**

In the last 24 hours (1 day)

In the last 25-48 hours (1 to 2 days ago)

In the last week (minimum 2 days ago, up to 7 days ago)

In the last fortnight (8-14 days ago)

**How would you describe your dietary preferences (standard diet, vegetarian, vegan, other?)**
