# Supplementary material for: Physical and psychological health in intern paramedics commencing shift work: Protocol for an exploratory longitudinal study
Source: PLoS One. 2022 Dec 1;17(12):e0273113. doi: 10.1371/journal.pone.0273113 (PMC9714933; doi:10.1371/journal.pone.0273113)
Supplement: S2 Appendix — (DOCX) [file pone.0273113.s002.docx]

**S2 Appendix. Shift work risk perception scale**

1. I worry about having poor health outcomes [Worry]

Strongly disagree – Disagree – Don’t disagree or agree – Agree – Strongly agree

2. I think my personal efforts will help control my risk of poor health [Self-efficacy]

Strongly disagree – Disagree – Don’t disagree or agree – Agree – Strongly agree

3. I’m at low risk for major illness [Perceived susceptibility/vulnerability]

Strongly disagree – Disagree – Don’t disagree or agree – Agree – Strongly agree

4. I am as healthy as anybody I know [Perceived health status]

Strongly disagree – Disagree – Don’t disagree or agree – Agree – Strongly agree

5. My working arrangements impact my health …

Very negatively – Negatively – Not at all – Positively – Very positively 
