# Supplementary material for: Physical and psychological health in intern paramedics commencing shift work: Protocol for an exploratory longitudinal study
Source: PLoS One. 2022 Dec 1;17(12):e0273113. doi: 10.1371/journal.pone.0273113 (PMC9714933; doi:10.1371/journal.pone.0273113)
Supplement: S3 Appendix — (DOCX) [file pone.0273113.s003.docx]

**S3 Appendix. Occupational trauma exposure semi-structed qualitative script**

*We’d like to ask you a few questions about being exposed to traumatic events.*

- Being a front line emergency service worker, you are going to be exposed to potentially traumatic events. Have you given any thought to how you will react to this exposure in terms of the support you might need?
- Are you aware of the support available in your organisation? Do you think you would be willing to access this support if you feel you need it? Why/why not?
- o you have informal support you think would be beneficial in this situation? What role would you like them to play?
- -Do you think your supervisor and colleagues have a role to play in helping you cope with this exposure? What can they do?
- If you find a traumatic event you are exposed to difficult to cope with how do you think your organisation will respond to help you?
