# Supplementary material for: Physical and psychological health in intern paramedics commencing shift work: Protocol for an exploratory longitudinal study
Source: PLoS One. 2022 Dec 1;17(12):e0273113. doi: 10.1371/journal.pone.0273113 (PMC9714933; doi:10.1371/journal.pone.0273113)
Supplement: S4 Appendix — (DOCX) [file pone.0273113.s004.docx]

**S4 Appendix. Sleep Inertia education experience semi-structed qualitative script** *I’d like to know if you know anything about sleep inertia and if you’ve received any formal training on this:*

- What do you know about sleep inertia?
- What information about sleep inertia have you received from your university training, if any?
- What information about sleep inertia have you received from your workplace, if any?
