# Supplementary material for: Physical and psychological health in intern paramedics commencing shift work: Protocol for an exploratory longitudinal study
Source: PLoS One. 2022 Dec 1;17(12):e0273113. doi: 10.1371/journal.pone.0273113 (PMC9714933; doi:10.1371/journal.pone.0273113)
Supplement: S5 Appendix — (DOCX) [file pone.0273113.s005.docx]

**S5 Appendix. Napping and sleep inertia during night shifts semi-structed qualitative script**

*I’d first like to get an idea of what you do and how you feel on night shifts:*

- Describe a typical night shift?
- During night shifts, when you are not actively working, what do you do?
- How do you feel in terms of alertness during night shifts?
- What do you do if you aren’t feeling alert or are feeling sleepy?
- If you have an opportunity to rest, what do you do?
- How often do you have opportunities to rest?
- How often do you utilise nap rooms/rest facilities?
- How often do your colleagues utilise nap rooms/rest facilities?

*I’d know like to understand more about what you experience or feel when waking from naps on night shifts.*

**Have you had any experience napping on night shift?**

- How do you feel immediately after waking up from a nap during break on night shift?
- How long do you feel it takes to feel fully alert after waking from a nap?
- How do you feel when you have to attend to an incident within minutes after waking from a nap?
- How does this feeling differ between high priority and low priority incidences?

Performance and Safety

- How do you feel about your job performance if you respond to an incident very soon after waking from a nap?
- How do you feel about your safety driving to an incident soon after waking from a nap?
- How do you feel about the safety of others around you when you are driving to an incident (this might include your co-workers or even other drivers on the road/pedestrians)

*Now that I know how you feel, I’d like to know more about what you actually do after waking from night time naps?*

- What do you typically do after waking from a nap if you do not have to attend to an incident right away?
- What do you typically do after waking from a nap if you have to attend an incident right away?
- If you feel sleepy after waking, what strategies do you use to make yourself feel more alert/awake?
- Are these methods effective? Why/why not?
- What strategies do your colleagues use?
- Does your workplace recommend any strategies?
- If you have a concern for your own safety or the safety of others, what do you do?
