# Supplementary material for: Physical and psychological health in intern paramedics commencing shift work: Protocol for an exploratory longitudinal study
Source: PLoS One. 2022 Dec 1;17(12):e0273113. doi: 10.1371/journal.pone.0273113 (PMC9714933; doi:10.1371/journal.pone.0273113)
Supplement: Attachment — Submitted filename: Response to Reviewer.docx [file pone.0273113.s006.docx]

**Response to Reviews:** Physical and psychological health in intern paramedics commencing shift work: Protocol for an exploratory longitudinal study

The authorship team wish to extend their thanks to the reviewer and editor for their review of our manuscript. Please find our responses below and throughout the revised manuscript.

| **Editorial Comments** | |
| --- | --- |
| We've received a single review that points to primarily issues either with method description, or with discussion of limitations of the method, mostly related to sampling. | Thank for your comments below. We hope that our changes have addressed the issues with methods and sampling. |
| It would be helpful for the authors to address with some detail the source population, approach to sampling (e.g. snowball, convenience etc) in a PICOT manner. | **We have added further information to the recruitment section**  “Recruitment will be conducted in collaboration with South Australian Ambulance Service. All interns undertaking SAAS internship orientation during the study recruitment period will be invited to participate in the project by a senior researcher (ACR) within two weeks of commencing intern training. ~~Interns will be invited by the senior researcher (ACR) to participate in the study when they commence their internship.~~ ACR will attend the training site during the intern orientation for each cohort, and provide a 30 minute presentation about the study. Following this presentation, interns will be able to ask any additional questions and sign up for the study if they choose. Participation is entirely voluntary, and intern paramedics are explicitly advised that participation is anonymous and will in no way impact their employment. Participants will only be recruited through the SAAS internship orientation, as outlined above.” |
| There are notable exclusions, and although explanations of exclusions aren’t necessarily standard elements of reporting checklists for survey studies, a description of the rationale behind these criteria would fit with quality rating tools for other article types and I think would help improve the quality of the paper. | **We have provided the following information to explain our choices for exclusion criteria**  “The exclusion of participants based on their ability to give informed consent is necessary given the sensitive health information collected during the research project. Participants who have worked for another ambulance agency for >12 months are not considered “new” paramedics and are therefore a different population, and are excluded. Finally, exclusion based on participants being unwilling or unable to commit to quarterly participation, ensures inclusion of participants who are able to contribute data which addresses the aims of the intended study.” |
| The reviewer also raises the Issue of a framing effect or bias. The disclosures are noted and as stated the primary funder is an independent entity with a public health remit. I think the authors could consider their framing with regards to the broad readership of PloS One, and aim to present a more neutral setup prior to establishing the case that they conclude from the data. This is not merely a semantic suggestion, since rating tools for assessed quality of prospective cohort studies include risk of bias assessments (e.g. [https://methods.cochrane.org/risk-bias-2](https://protect-au.mimecast.com/s/5vunC71ZvqhwlM1li2VByO?domain=methods.cochrane.org)). | **Thank you for your feedback.**  **We hope we have addressed the reviewers concerns through the addition of the follow details and references throughout to highlight the possible positive changes and to better frame as an unbiased cohort study. We have also removed any unnecessary language that may appear biased.**  “In addition to what is currently known about health outcomes in shift workers more broadly, evidence suggests that paramedics experience high levels of stress, depression, fatigue, burnout and increased risk of post-traumatic stress disorder[19-23]. A recent systematic review suggests that organisational support and individual coping mechanisms may protect against the development of these mental health risks in paramedics [24]. There is a need to understand psychosocial factors that may support good health during the transition to shift work. Collectively, there is a lack of longitudinal studies that investigate changes, positive or negative, in physical and psychological health outcomes in paramedics, particularly when commencing their career[25], which makes it challenging to understand the temporality of these experiences.  ~~Despite evidence that shift work exposure increases the prevalence of adverse health outcomes and risk of mortality in the medium- to long-term, it is not feasible to dispense with shift work practices, particularly in the context of emergency health care where demands are constant and unpredictable. Therefore, a~~ A better understanding of the effects of shift work on a paramedic’s physical and psychological health is needed to determine associations with medium to long term health outcomes and if necessary, assist in early, preventive strategies to minimise adverse impacts of shift work on health and wellbeing. ~~harm.~~”  **We have made the following changes to our discussion:**  **“**Shift work is associated with negative health outcomes. ~~However,~~ Our understanding of possible underlying mechanisms for or protective factors against these medium- to long-term health outcomes, and the possible onset of these mechanisms during the transition to shift work are not well understood. This means we have no clear understanding of the sequalae of health changes, positive or negative, which impacts our ability to provide preventive advice and develop strategies for early career shift workers.”  **We have also changed our conclusion to better reflect our intention to find all changes, positive or negative.**  “The proposed study will allow for better understanding of biopsychosocial changes during the transition to shift work. By conducting this exploratory, observational study we may better understand if any changes, positive or negative, occur during early career shift work. ~~And thus, identify where future research, policy and practical intervention may provide benefit to better mitigate the negative medium- to long-term health outcomes associated with shift work.~~”  **We feel that our research aims, hypothesis and research questions present as unbiased.** |
| **Reviewer 1** | |
| Thanks for the opportunity to review and apologies for the delay. Congratulations on your protocol on this essential and currently increasingly evidenced area. | Thank you for your positive feedback |
| The aims and objectives are clearly defined. A hypothesis should be provided. | **We have added the following hypothesis:**  **Hypothesis**  It is hypothesised that the transition to shift work will be associated with changes in cardiometabolic risk, the gut microbiome, health behaviours and wellbeing, when compared to baseline. |
| The risk of selection bias is always present in these types of studies, particularly when participation is voluntary. I do not see a way around this, but worth considering in the analysis/discussion. | **We have added the following to the Statistical Analysis section:**  “Key descriptive statistics (e.g. demographics, sleep and health at baseline) will be compared between participants lost to follow-up and those who continued within the study, using t-test or non-parametric equivalent if necessary, to determine any factors which differ between the two groups.”  **We have added the following to our discussion:**  “The present study will be susceptible to selection bias. In order to best mitigate this, all eligible interns during the recruitment period will be offered the opportunity to participate, and we will transparently report any baseline differences in those who are lost to follow-up, compared to those who remain in the study. We will not be able to compare differences in those who did not consent to the study, as we do not have data on the interns not included in the present study. This risk of bias will be acknowledged in any reporting of findings from the study to provide context.” |
| The columns in table 1 should be changed to the actual times, not just T0,1,2,3,4 | We have now changed these to the timepoints. |
| The caption in table 1 is to be moved to methods. | We have now moved this caption to the methods under “Cardiometabolic risk factors” |
| Table 2 lists the surveys to be used. This seems excessive and may result in high drop-out rates due to survey fatigue. Consideration about consolidating this list will likely lead to a more complete, although truncated data set. | We thank the reviewer for their comment. Unfortunately, as recruitment as began for this project we are unable to change the amount of surveys. We will however consider this for future studies in the area. |
| The statistical analysis plan lacks details. | **We have added further detail to the statistical analysis section:**  Analysis will be conducted to investigate any changes in i) cardiometabolic risk factors, ii) human gut microbiome, iii) health behaviours (e.g. physical activity, alcohol, smoking, dietary intake) and sleep, and iv) ~~possible psychosocial factors associated with~~ wellbeing during first year of shift work. Missing data will be addressed using multiple imputation, as appropriate. Descriptive statistics will be included to provide information regarding participant characteristics. To examine any changes over time, ~~As appropriate for the questions of interest,~~ statistical modelling will be conducted using mixed effects regression, specifying a random effect of subject on the intercept, allowing interns to vary according to individual baseline levels, as well as tracking progress over time, appropriately accounting for serial correlation. Effect sizes and 95% confidence intervals will be considered during analyses. Key descriptive statistics (e.g. demographics, sleep and health at baseline) will be compared between participants lost to follow-up and those who continued within the study, using t-test or non-parametric equivalent if necessary, to determine any factors which differ between the two groups. |
| Reading the paper, I get the sense that the authors want to demonstrate the extent of the negative impact of shift work. Rather they could take a more unbiased approach and aim to determine how commencing shift work impacts health in any direction. | **Thank you for your feedback.**  **We hope we have addressed the reviewers concerns through the addition of the follow details and references throughout to highlight the possible positive changes and to better frame as an unbiased cohort study. We have also removed any unnecessary language that may appear biased.**  “In addition to what is currently known about health outcomes in shift workers more broadly, evidence suggests that paramedics experience high levels of stress, depression, fatigue, burnout and increased risk of post-traumatic stress disorder[19-23]. A recent systematic review suggests that organisational support and individual coping mechanisms may protect against the development of these mental health risks in paramedics [24]. There is a need to understand psychosocial factors that may support good health during the transition to shift work. Collectively, there is a lack of longitudinal studies that investigate changes, positive or negative, in physical and psychological health outcomes in paramedics, particularly when commencing their career[25], which makes it challenging to understand the temporality of these experiences.  ~~Despite evidence that shift work exposure increases the prevalence of adverse health outcomes and risk of mortality in the medium- to long-term, it is not feasible to dispense with shift work practices, particularly in the context of emergency health care where demands are constant and unpredictable. Therefore, a~~ A better understanding of the effects of shift work on a paramedic’s physical and psychological health is needed to determine associations with medium to long term health outcomes and if necessary, assist in early, preventive strategies to minimise adverse impacts of shift work on health and wellbeing. ~~harm.~~”  **We have made the following changes to our discussion:**  **“**Shift work is associated with negative health outcomes. ~~However,~~ Our understanding of possible underlying mechanisms for or protective factors against these medium- to long-term health outcomes, and the possible onset of these mechanisms during the transition to shift work are not well understood. This means we have no clear understanding of the sequalae of health changes, positive or negative, which impacts our ability to provide preventive advice and develop strategies for early career shift workers.”  **We have also changed our conclusion to better reflect our intention to find all changes, positive or negative.**  “The proposed study will allow for better understanding of biopsychosocial changes during the transition to shift work. By conducting this exploratory, observational study we may better understand if any changes, positive or negative, occur during early career shift work. ~~And thus, identify where future research, policy and practical intervention may provide benefit to better mitigate the negative medium- to long-term health outcomes associated with shift work.~~”  **We feel that our research aims, hypothesis and research questions present as unbiased.** |
